# A Comparative Study of Oral Tofacitinib and Oral Methotrexate in the Treatment of Patients With Moderate to Severe Chronic Plaque Psoriasis

**DOI:** 10.7759/cureus.103264

**Published:** 2026-02-09

**Authors:** Pooja Unnikrishnan, Kirankanth Vudayana, Dilipchandra Chintada, Mohammed Khatija Begum, Sai Sriya Chalamalasetty, Pallavi Gullipalli

**Affiliations:** 1 Dermatology, Venereology and Leprosy, Great Eastern Medical School and Hospital, Srikakulam, IND

**Keywords:** chronic plaque psoriasis, janus kinase inhibitors, methotrexate, psoriasis area and severity index, tofacitinib

## Abstract

Background

Chronic plaque psoriasis is a common immune-mediated dermatosis that often requires long-term systemic therapy in patients with moderate to severe disease. Methotrexate has been a cornerstone of systemic treatment for decades; however, concerns regarding delayed onset of action, cumulative toxicity, and the need for close laboratory monitoring have driven interest in newer targeted oral therapies. Tofacitinib, an oral Janus kinase (JAK) inhibitor, modulates multiple cytokine signaling pathways implicated in psoriasis pathogenesis, though comparative data from the Indian population remain limited.

Objectives

The present study aimed to compare the efficacy, time to response, relapse rates, and safety profile of oral tofacitinib versus oral methotrexate in patients with moderate to severe chronic plaque psoriasis.

Methods

This prospective, randomized, open-label comparative study was conducted over 18 months (August 2023 to January 2025) at Great Eastern Medical School & Hospital, a tertiary care teaching hospital in South India. Adult patients with biopsy-proven chronic plaque psoriasis of at least three months’ duration, a Psoriasis Area and Severity Index (PASI) score greater than 10, and body surface area involvement exceeding 10% were enrolled. Forty-two eligible patients were randomized into two groups: Group A (n = 21) received oral tofacitinib 5 mg twice daily, while Group B (n = 21) received oral methotrexate 10 mg once weekly with folic acid supplementation. Clinical assessment using PASI was performed at baseline and at two, four, eight, 12, and 16 weeks. Treatment efficacy, PASI 75 and PASI 90 responses, time to PASI 75, relapse, and adverse events were analyzed.

Results

Both treatment groups demonstrated a progressive and statistically significant reduction in mean PASI scores over the 16-week treatment period, with no statistically significant difference in mean PASI reduction between the two groups at individual follow-up visits. Tofacitinib showed a faster onset of action, with a higher proportion of patients achieving PASI 75 by week 12 (57.1%), whereas methotrexate demonstrated a higher cumulative PASI 75 response by week 16 (71.4%). Achievement of PASI 90 at week 16 was significantly higher in the tofacitinib group compared to the methotrexate group (57.1% vs. 19.0%; p < 0.05). Relapse was observed more frequently in the methotrexate group, although this difference did not reach statistical significance. Mild elevation of liver enzymes was the most commonly observed adverse effect in both groups, and no serious adverse events were recorded.

Conclusion

Oral tofacitinib and methotrexate are both effective systemic therapies for moderate to severe chronic plaque psoriasis. Tofacitinib offers the advantage of faster and deeper clinical clearance, while methotrexate demonstrates comparable efficacy over a longer treatment duration. Tofacitinib may be considered a useful oral alternative in patients requiring rapid disease control.

## Introduction

Chronic plaque psoriasis is a chronic immune-mediated inflammatory skin disorder characterized by well-demarcated erythematous plaques with silvery scales and a relapsing-remitting course. The global prevalence of psoriasis ranges from 0.1% to 3%, with Indian studies reporting prevalence between 0.44% and 2.8% [1,2]. Psoriasis is now recognized as a systemic inflammatory disease associated with multiple comorbidities, including psoriatic arthritis, metabolic syndrome, cardiovascular disease, and significant impairment in quality of life [3-5].

Systemic therapy is often required for the management of moderate to severe chronic plaque psoriasis. Methotrexate has long been considered a cornerstone systemic agent because of its proven efficacy, affordability, and widespread availability [6]. However, its use is limited by delayed onset of action, cumulative hepatotoxicity, myelosuppression, and the requirement for regular laboratory monitoring [7-9]. Advances in the understanding of psoriasis pathogenesis have highlighted the role of key cytokines such as tumor necrosis factor-α, interleukin-17, and interleukin-23, along with downstream activation of the Janus kinase-signal transducer and activator of transcription (JAK-STAT) pathway [10,11]. These insights have led to the development of newer targeted oral therapies.

Tofacitinib is an oral JAK inhibitor that primarily inhibits JAK1 and JAK3, thereby modulating multiple cytokine signaling pathways involved in psoriasis pathophysiology [12]. Phase II and III randomized controlled trials have demonstrated its efficacy in achieving PASI 75 and PASI 90 responses in patients with moderate to severe chronic plaque psoriasis [13-15]. Meta-analyses and systematic reviews have further supported its short-term efficacy and acceptable safety profile, although long-term safety remains under evaluation [16-18].

Despite increasing global evidence, head-to-head comparative studies between tofacitinib and methotrexate remain limited, particularly in the Indian population, where treatment decisions are influenced by affordability, accessibility, and patient preference [19-22]. The present study was therefore undertaken to compare the efficacy, safety, relapse rates, and time to response of oral tofacitinib versus oral methotrexate in patients with moderate to severe chronic plaque psoriasis.

## Materials and methods

This prospective, randomized, open-label comparative study was conducted over an 18-month period (August 2023 to January 2025) at Great Eastern Medical School & Hospital, a tertiary care teaching hospital in South India. Ethical approval was obtained from the Institutional Ethics Committee of Great Eastern Medical School & Hospital, Ragolu, Srikakulam, Andhra Pradesh, India (IEC Registration No.: 89/IEC/GEMS&H/2023; DHR Registration No.: EC/NEW/INST/2021/2044; CDSCO Registration No.: ECR/1521/Inst/AP/2021) prior to study initiation, and written informed consent was obtained from all participants. The study was conducted in accordance with the principles of the Declaration of Helsinki.

All patients attending the dermatology outpatient department with suspected chronic plaque psoriasis during the study period were screened for eligibility. A total of 68 patients were screened, of whom 42 fulfilled the inclusion criteria and were enrolled. Eligible participants were adults aged 18 years or older with clinically and histopathologically confirmed chronic plaque psoriasis of at least three months’ duration, a PASI score greater than 10, and body surface area involvement exceeding 10%. Patients with involvement of the face, scalp, groin, hands, and feet were included if the severity criteria were met.

Patients with other variants of psoriasis such as guttate, pustular, or erythrodermic psoriasis, other inflammatory dermatoses, extensive scarring or pigmentation interfering with assessment, active or latent infections, significant hepatic, renal, or hematological abnormalities, pregnancy or lactation, history of malignancy, known hypersensitivity to methotrexate or tofacitinib, or prior use of biologic agents or JAK inhibitors within the preceding six months were excluded.

Randomization was performed using computer-generated random numbers, and allocation concealment was ensured using sealed opaque envelopes. Twenty-one patients were allocated to Group A and received oral tofacitinib 5 mg twice daily. Twenty-one patients were allocated to Group B and received oral methotrexate 10 mg once weekly, along with folic acid 5 mg once weekly, administered 24 hours after methotrexate. No additional systemic antipsoriatic drugs were permitted during the study. Individual patient treatment and follow-up duration were 16 weeks.

Baseline demographic details, disease duration, prior treatment history, and presence of psoriatic arthritis were recorded. Disease severity was assessed using PASI at baseline and at weeks 2, 4, 8, 12, and 16. Treatment efficacy was evaluated by change in the mean PASI score, proportion of patients achieving PASI 75 and PASI 90, time to achieve PASI 75, and relapse. Relapse was operationally defined as loss of PASI 75 or a ≥50% increase in PASI from the lowest achieved score. Safety assessment was carried out throughout the study period.

Baseline and follow-up laboratory investigations included complete blood count, liver function tests, renal function tests, lipid profile, and screening for viral hepatitis and tuberculosis as per institutional protocol.

Statistical analysis was performed using the IBM SPSS Statistics for Windows, Version 25.0 (released 2017, IBM Corp., Armonk, NY). Continuous variables were expressed as mean ± standard deviation and compared using an independent samples t-test, while categorical variables were expressed as frequencies and percentages and compared using a chi-square test. Baseline comparability between groups was assessed using appropriate statistical tests. A p-value less than 0.05 was considered statistically significant.

## Results

Demographic and clinical characteristics

A total of 42 patients with moderate to severe chronic plaque psoriasis were enrolled and randomized equally into two treatment groups, with 21 patients in the tofacitinib group (Group A) and 21 patients in the methotrexate group (Group B). The mean age of patients in Group A was comparable to that in Group B, and there was no statistically significant difference in age or sex distribution between the two groups (Table 1). The mean duration of disease, baseline PASI scores, body surface area involvement, and the proportion of patients with associated psoriatic arthritis were also comparable at baseline, indicating adequate randomization. Baseline demographic and clinical parameters were comparable between the two groups (p > 0.05).

**Table 1:** Baseline demographic and clinical characteristics of the study participants (N = 42).

Variable	Category	n (%) / Mean ± SD
Age (years)	Mean ± SD (Range)	46.59 ± 13.17 (18–72)
	<20	1 (2.4)
	20–30	4 (9.5)
	30–40	10 (23.8)
	40–50	8 (19.0)
	50–60	11 (26.2)
	>60	8 (19.0)
Sex	Male	32 (76.2)
	Female	10 (23.8)
Duration of disease	2–3 years	6 (14.3)
	4–5 years	7 (16.7)
	>5 years	29 (69.0)
Site of involvement	Extensive (trunk ± scalp ± limbs)	18 (42.9)
	Trunk + limbs	14 (33.3)
	Scalp + trunk + limbs	7 (16.7)
	Localized involvement	3 (7.1)
Nail involvement	Present	34 (81.0)
	Absent	8 (19.0)
Psoriatic arthritis	Present	2 (4.8)
	Absent	40 (95.2)
Previous treatment	None	13 (31.0)
	Topical steroids	13 (31.0)
	Oral/topical (unknown)	16 (38.0)

Change in PASI scores during follow-up

**Figure 1:**
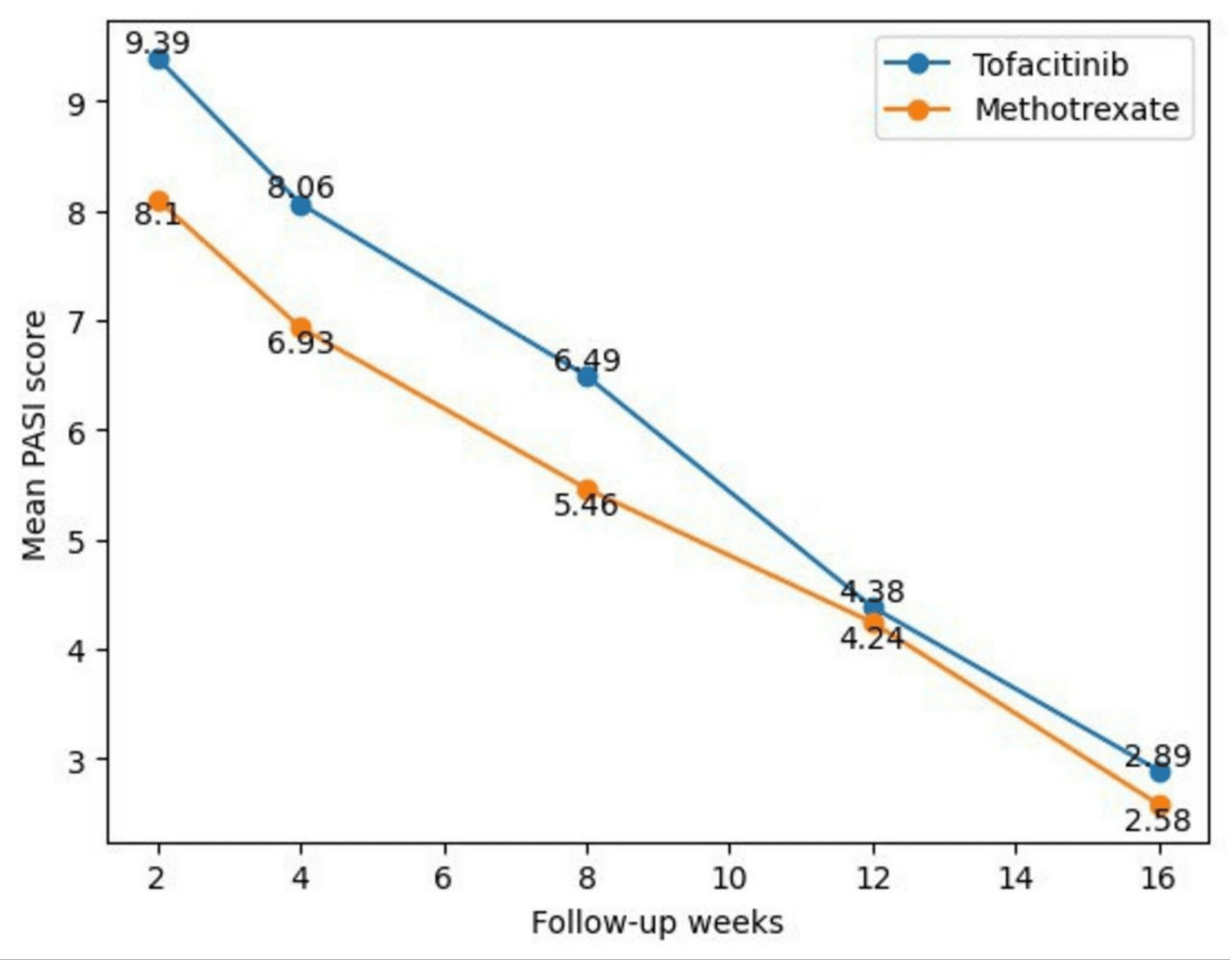
Comparison of mean Psoriasis Area and Severity Index (PASI) score reduction between the oral tofacitinib and oral methotrexate groups during the study period.

Both treatment groups showed a progressive and statistically significant reduction in mean PASI scores from the baseline through week 16. In the tofacitinib group, a rapid decline in PASI score was observed as early as week 4, whereas in the methotrexate group, PASI reduction was more gradual (Figure 1). At each follow-up visit (weeks 2, 4, 8, 12, and 16), the mean PASI score was lower than baseline in both groups. However, there was no statistically significant difference in the mean PASI score reduction between the two groups at individual follow-up visits (Table 2).

**Table 2:** Comparison of mean Psoriasis Area and Severity Index (PASI) scores between the two groups during the study period (independent samples t-test).

Follow-up period	Group	N	Mean PASI ± SD	Median	t-value	p-value
Week 2	Group A (Tofacitinib)	21	9.39 ± 3.53	9.8	1.28	0.206
	Group B (Methotrexate)	21	8.10 ± 2.97	6.8		
Week 4	Group A (Tofacitinib)	21	8.06 ± 2.87	8.1	1.32	0.200
	Group B (Methotrexate)	21	6.93 ± 2.68	5.6		
Week 8	Group A (Tofacitinib)	21	6.49 ± 2.38	6.5	1.51	0.140
	Group B (Methotrexate)	21	5.46 ± 2.02	4.6		
Week 12	Group A (Tofacitinib)	21	4.38 ± 1.66	4.6	0.27	0.790
	Group B (Methotrexate)	21	4.24 ± 1.49	3.8		
Week 16	Group A (Tofacitinib)	21	2.89 ± 1.13	2.8	1.00	0.320
	Group B (Methotrexate)	21	2.58 ± 0.89	2.5		

PASI 75 and PASI 90 response

**Figure 2:**
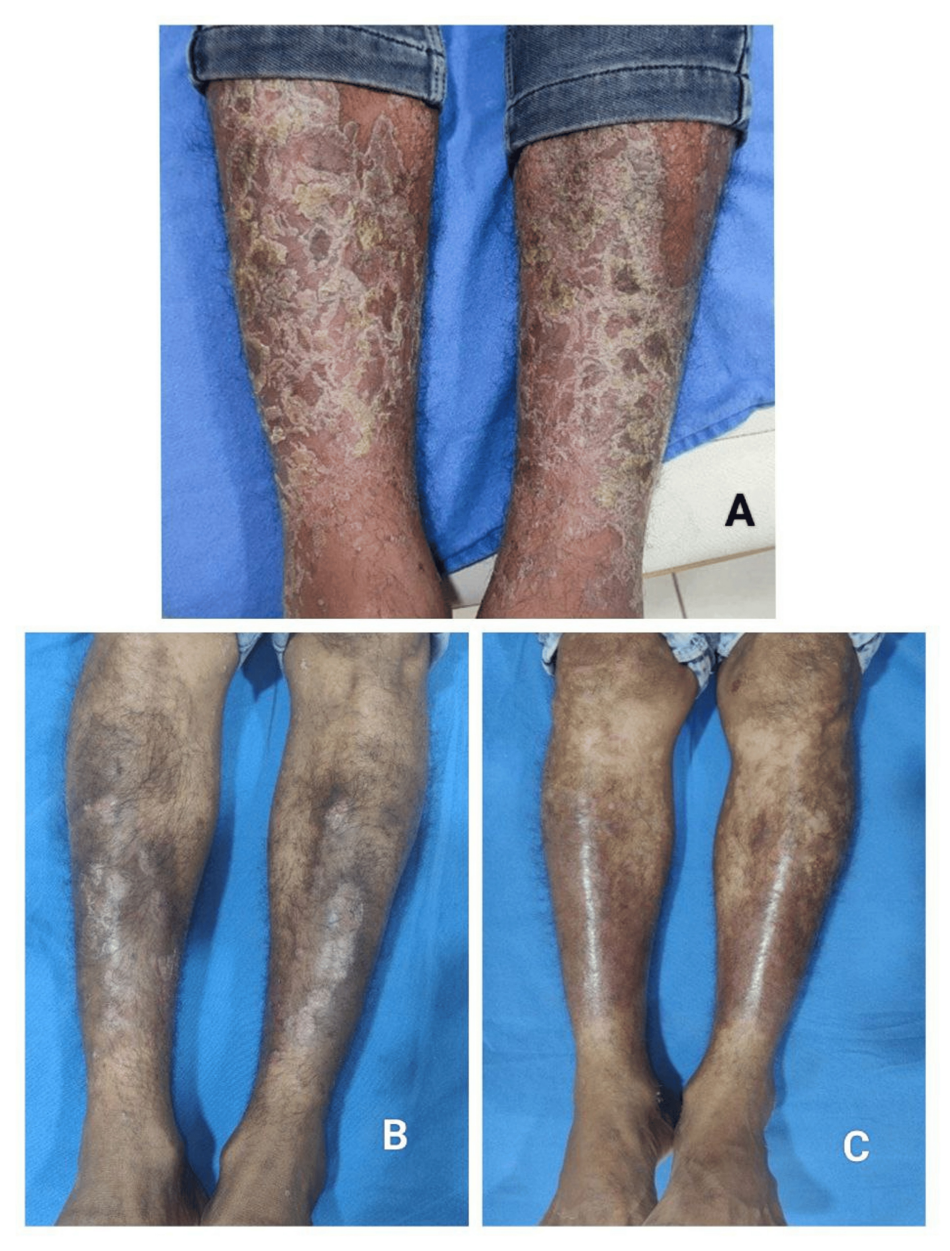
Clinical photographs showing response to oral tofacitinib in chronic plaque psoriasis. (A) Baseline photograph showing multiple well-defined erythematous plaques with thick adherent silvery scales over the bilateral lower legs prior to initiation of treatment. (B) Follow-up photograph at eht weeks demonstrating marked reduction in erythema, scaling, and plaque thickness following oral tofacitinib therapy. (C) Follow-up photograph at 16 weeks showing near-complete clearance of lesions with residual post-inflammatory hyperpigmentation.

**Figure 3:**
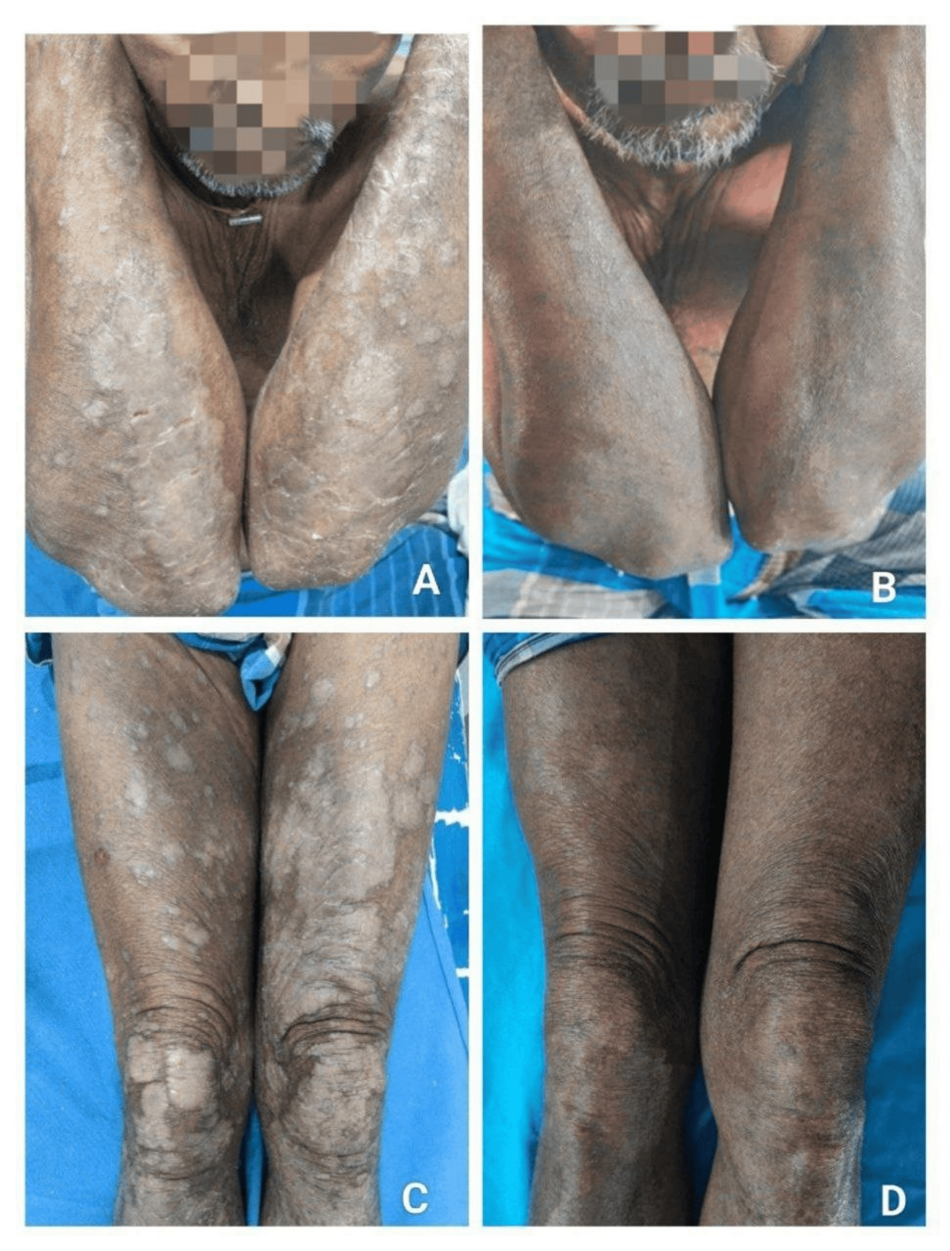
Clinical photographs showing response to oral methotrexate in chronic plaque psoriasis. (A) Baseline photograph showing extensive, well-defined erythematous plaques with thick scaling over the extensor surfaces of the upper limbs prior to initiation of methotrexate therapy. (B) Follow-up photograph at 16 weeks demonstrating reduction in erythema, scaling, and plaque thickness following treatment. (C) Baseline photograph showing multiple chronic plaque lesions over the bilateral lower limbs before treatment. (D) Follow-up photograph at 16 weeks showing marked clinical improvement with near-complete resolution of plaques and residual post-inflammatory hyperpigmentation after oral methotrexate therapy.

By week 12, a higher proportion of patients in the tofacitinib group achieved PASI 75 compared to the methotrexate group. By week 16, PASI 75 response was observed in 71.4% of patients in the methotrexate group and 66.7% of patients in the tofacitinib group, with no significant statistical difference between the groups. Achievement of PASI 90 at week 16 was significantly higher in the tofacitinib group compared to the methotrexate group, indicating a greater proportion of near-complete clearance with tofacitinib (Figures 2, 3).

Time to achieve PASI 75

Patients treated with tofacitinib achieved PASI 75 earlier than those treated with methotrexate. The majority of PASI 75 responders in the tofacitinib group achieved this outcome by week 12, whereas a substantial proportion of patients in the methotrexate group required up to 16 weeks to reach PASI 75. (Figures 2, 3). Patients treated with tofacitinib achieved PASI 75 earlier; however, as a formal time-to-event analysis was not performed, this observation is interpreted cautiously.

Relapse during follow-up

**Figure 4:**
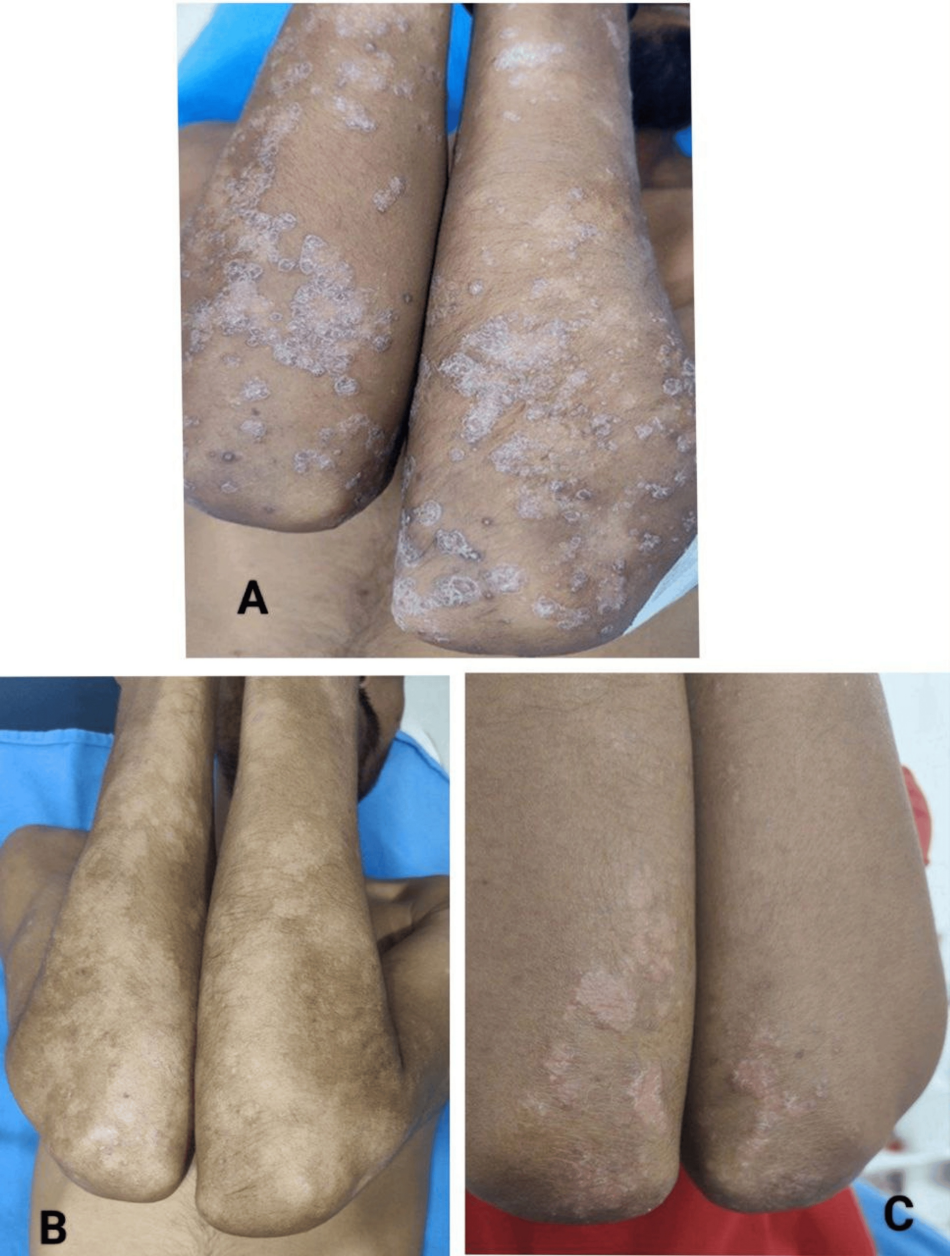
Clinical photographs showing relapse of chronic plaque psoriasis following methotrexate therapy. (A) Baseline photograph showing multiple well-defined erythematous plaques with thick adherent silvery scales over the extensor surfaces of the elbows prior to initiation of methotrexate treatment. (B) Follow-up photograph after 12 weeks of completion of methotrexate therapy showing marked clinical improvement with significant reduction in erythema, scaling, and plaque thickness. (C) Follow-up photograph during post-treatment period (after 14 weeks) demonstrating relapse, characterized by reappearance of erythematous scaly plaques over the extensor surfaces.

Relapse is defined as a loss of PASI 75 or a ≥50% increase in PASI from the lowest achieved score. Relapse after initial response in PASI was observed more frequently in the methotrexate group compared to the tofacitinib group during the follow-up period. However, this difference did not reach statistical significance (Figure 4).

Safety and adverse events

Both treatment regimens were generally well tolerated. Mild elevation of liver enzymes of AST/ALT (<2× ULN) was the most commonly observed adverse event in both groups. Mild, non-clinically significant lipid elevations were noted in some patients receiving tofacitinib. No serious adverse events, severe infections, or treatment-limiting toxicities were recorded during the study period. No patient required permanent discontinuation of therapy due to adverse effects.

## Discussion

In this randomized comparative study, both oral tofacitinib and oral methotrexate demonstrated significant clinical efficacy in patients with moderate to severe chronic plaque psoriasis, as reflected by progressive reductions in PASI scores. These findings are consistent with earlier studies that have established methotrexate as an effective conventional systemic therapy and tofacitinib as an efficacious targeted oral agent in psoriasis management [6,13]. The comparable reduction in mean PASI scores between the two groups aligns with previous real-world and clinical studies evaluating systemic therapies for psoriasis.

A relatively faster onset of clinical response was observed with tofacitinib, with a higher proportion of patients achieving PASI 75 by week 12. Similar early response patterns have been reported in phase III trials by Papp et al. and Bissonnette et al., where rapid PASI improvement was documented within the first eight to 12 weeks of therapy [14,15]. By contrast, methotrexate demonstrated a more gradual improvement, with higher cumulative PASI 75 response rates observed at later follow-up, a response pattern that has been consistently described in Indian and international studies by Dogra et al. and Warren et al. [7,8].

Achievement of PASI 90, indicative of near-complete disease clearance, was significantly higher in the tofacitinib group. This finding is comparable to results reported in randomized trials and pooled analyses by Valenzuela et al. and Armstrong et al., which demonstrated higher PASI 90 response rates with tofacitinib compared to conventional systemic agents [15-17]. Lower PASI 90 response rates with methotrexate, particularly at lower weekly doses, have been reported in previous studies and may account for the observed differences in deeper clearance between the two treatments [6-9].

Relapse was observed less frequently in patients treated with tofacitinib, although the difference did not reach statistical significance. Long-term extension studies by Valenzuela et al. have reported sustained disease control with continued tofacitinib therapy, while methotrexate-treated patients have been noted to experience relapse following dose reduction or treatment interruption [18,23]. The relapse patterns observed in the present study are therefore in agreement with trends reported in existing literature.

The safety profile observed in this study was comparable to that reported in earlier clinical trials and observational studies. Mild elevations of liver enzymes were the most commonly observed adverse events in both treatment groups, consistent with findings reported by Warren et al. and Papp et al. [9,14]. Mild, non-clinically significant lipid elevations observed with tofacitinib have also been described in previous studies and did not necessitate treatment discontinuation [14,18]. Improvement in joint symptoms among patients with concomitant psoriatic arthritis treated with tofacitinib is consistent with randomized trials by Mease et al., supporting its dual efficacy in cutaneous and articular manifestations of psoriatic disease [24,25].

Overall, when compared with existing literature, the findings of the present study reinforce evidence that tofacitinib offers a faster onset of action and a greater likelihood of achieving higher levels of clearance, while methotrexate remains a reliable and widely used systemic therapy with established efficacy. These results add meaningful comparative data from an Indian population and are particularly relevant to routine clinical practice where individualized treatment decisions are required [26].

Limitations of the study

This study has certain limitations. The relatively small sample size and single-center design limit the generalizability of the findings. The short duration of treatment and follow-up restricts evaluation of long-term efficacy, safety, and relapse rates. The open-label nature of the study may have introduced observer or reporting bias. In addition, baseline differences such as sex distribution and previous treatment exposure between the groups could have influenced treatment outcomes. Lastly, adverse event assessment was limited and may not have captured rare or delayed side effects.

## Conclusions

The present randomized comparative study demonstrates that both oral tofacitinib and oral methotrexate are effective systemic therapies for moderate to severe chronic plaque psoriasis, resulting in significant clinical improvement as evidenced by progressive reductions in PASI scores. Tofacitinib showed a more rapid onset of action with earlier achievement of PASI 75 and a higher proportion of patients attaining PASI 90, indicating deeper and faster disease clearance. Methotrexate, while effective, exhibited a comparatively slower response and was associated with a higher tendency for relapse during follow-up. The safety profiles of both drugs were acceptable, with no serious adverse events observed, although methotrexate required more intensive laboratory monitoring.

These findings highlight the potential role of tofacitinib as a valuable oral alternative to conventional systemic therapy, particularly in patients requiring rapid disease control, higher levels of clearance, or those with associated psoriatic arthritis. Methotrexate continues to remain a cost-effective and reliable option, especially in resource-limited settings; however, the risk of relapse and cumulative toxicity should be carefully considered during long-term management. Larger multicentric studies with further follow-up are warranted to further evaluate long-term efficacy, relapse patterns, and safety outcomes. Overall, this study adds meaningful comparative evidence from an Indian population and supports individualized treatment selection based on disease severity, comorbidities, and patient preference.
